# HIF-1α contributes to hypoxia adaptation of the naked mole rat

**DOI:** 10.18632/oncotarget.22767

**Published:** 2017-11-30

**Authors:** Bang Xiao, Shiyong Wang, Guoshi Yang, Xiaoxi Sun, Shanmin Zhao, Lifang Lin, Jishuai Cheng, Wenjing Yang, Wei Cong, Wei Sun, Guanghan Kan, Shufang Cui

**Affiliations:** ^1^ Laboratory Animal Centre, The Second Military Medical University, Shanghai 200433, China; ^2^ Division of Teaching Support, Training Department, The Second Military Medical University, Shanghai 200433, China; ^3^ China Astronaut Research and Training Center, Beijing 100094, China

**Keywords:** HIF-1α, VEGFA, naked mole rat, hypoxic adaptation

## Abstract

**Background/Aims:**

Naked mole rats (NMRs) spend their lives in burrow systems containing very low levels of oxygen, indicating long-term hypoxic exposure, and suggesting that pathological changes caused by hypoxia are attenuated or absent in this hypoxia-tolerant species. The mechanisms underlying NMRs hypoxia tolerance remain poorly understood. In this study, we explored whether hypoxia inducible factor 1α (HIF-1α), and vascular endothelial growth factor A (VEGFA) play a role in NMRs adaption to hypoxia.

**Methods:**

Primary hepatic stellate cells (HSCs) isolated from NMRs and mice were treated with 50 μM YC-1, 50 μM KC7F2 or VEGFA siRNA. HIF-1α or VEGFA expression was detected by Western blot and real-time PCR. Apoptosis was determined by flow cytometry. The expression of autophagy markers (LC3 and p62) was detected by Western blot.

**Results:**

Our results showed that HIF-1α and VEGFA expression in NMRs was significantly higher than in hypoxia-sensitive mice. Inhibition of HIF-1α expression induced apoptosis in both NMR and mouse HSCs following hypoxia. However, blocking VEGFA transcription results in a significant increase of apoptosis in both NMR and mouse HSCs before and after hypoxia. In addition, NMR HSCs displayed higher levels of autophagy (ratio of LC3ΙΙ/LC3Ι = 9.6) than mouse HSCs (relative ratio of LC3ΙΙ/ LC3Ι = 4.9) under hypoxic conditions.

**Conclusion:**

We conclude that HIF-1α activation may be an important mechanism for hypoxia adaption. However, high expression of VEGFA follows HIF-1α activation in NMRs.

## INTRODUCTION

Naked mole rats (Heterocephalus glaber) are a small rodent species that live underground in Kenya, Ethiopia and Somalia. This strictly subterranean rodent tolerates hypoxia, hypercapnia, and soil-based toxins [1]. It thrives in extreme environments for as much as 30 years, exceeding the life span of any other rodents of similar size [2]. Under experimental conditions, naked mole-rats tolerate hours of extreme hypoxia and survive 18 min of total oxygen deprivation (anoxia) without apparent injury [3]. Therefore NMRs are naturally tolerant of hypoxia, and provide an excellent model for studying adaptation to chronic hypoxia.

Previous studies found that, compared with other underground mammals, NMRs have evolved adaptive strategies to cope with chronic hypoxia in their burrows. For example, NMRs reduce their basal metabolic rates to adapt to their hypoxic environment [4]. NMRs can also adapt to the low oxygen environment by changing their physiological characteristics [5].

Previous studies have identified hypoxia inducible factor 1 (HIF-1) as the master regulator of mammalian O_2_ homeostasis [6]. HIF-1 activates transcription of target genes by binding to their promoters, initiating physiological and biochemical responses to hypoxic stress that reduce or eliminate the negative impact caused by low oxygen. Vascular endothelial growth factor (VEGF) is a powerful angiogenic factor critical in response to tissue hypoxia. VEGF expression is regulated by HIF-1 under hypoxic conditions [7]. HIF-1α is the hypoxia-inducible subunit, and is not present under normoxia; its expression is regulated at both the transcriptional and posttranscriptional stages. HIF-1β is the constitutive subunit and combines with HIF-1α to form a heterodimeric transcription factor that binds to the 3′ enhancer region of target genes to activate transcription [8]. Genome sequencing shows that, compared with other mammals, the NMRs HIF-1α gene may avoid degradation through ubiquitination by specifically exchanging amino acids in the binding site of VHL [9]. Therefore, we speculated that NMRs reduce damage caused by hypoxia by constitutively high HIF-1α levels, leading to increased VEGFA transcription.

In this study, we compared HIF-1α and VEGFA expression in tissues from adult NMRs and mice, and evaluated the levels of apoptosis in NMR or mouse HSCs treated with a specific HIF-1α inhibitor (YC-1 or KC7F2) or VEGFA-siRNA before and after hypoxic exposure.

## RESULTS

### HIF-1α and VEGF expression is significantly higher in NMRs than in mice

**Figure 1:**
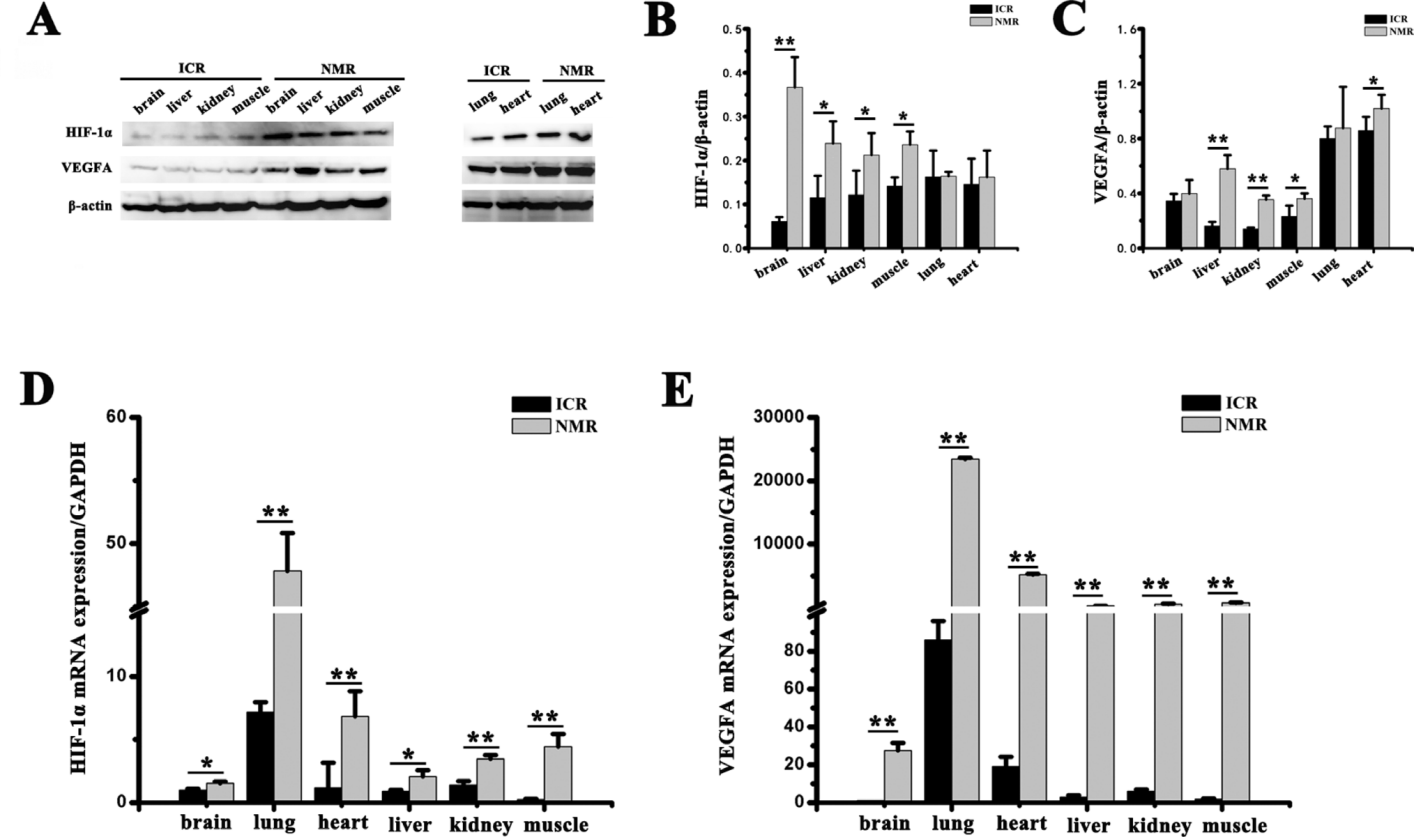
Expression of HIF-1α and VEGFA in brain, lung, heart, liver, kidney and muscle (**A**) HIF-1α and VEGFA expression in brain, lung, heart, liver, muscle and kidney from adult NMRs (NMRs, 8 months old) and adult ICR mice (ICR, 8 weeks old) was detected by Western blot. β-Actin was used as an internal loading control. Band density analysis of (**B**) HIF-1α and (**C**) VEGFA. Real-time PCR analysis of (**D**) HIF-1α and (**E**) VEGFA mRNA expression in brain, heart, lung, liver, kidney and muscle from NMRs and mice. GAPDH was used as an internal reference. The data represent means ± SEM of triplicate measurements obtained from three different animals of each species. ^*^p < 0.05, ^**^p < 0.01.

Western blot results showed that compared with mice, NMRs had higher levels of HIF-1α or VEGFA in brain, liver, muscle and kidney (*p* < 0.05). Brain tissue from mice and NMRs had high levels of VEGFA (Figure 1). Real-time PCR showed that brain, heart, lung, liver, kidney and muscle from NMRs had significantly higher expression of HIF-1α and VEGFA mRNA than mice (*p* < 0.05, Figure 1D, 1E). These results showed that NMRs maintained higher levels of HIF-1α and VEGFA than mice.

### HIF-1α and VEGFA expression in tissues of NMRs exposed to acute hypoxia

**Figure 2:**
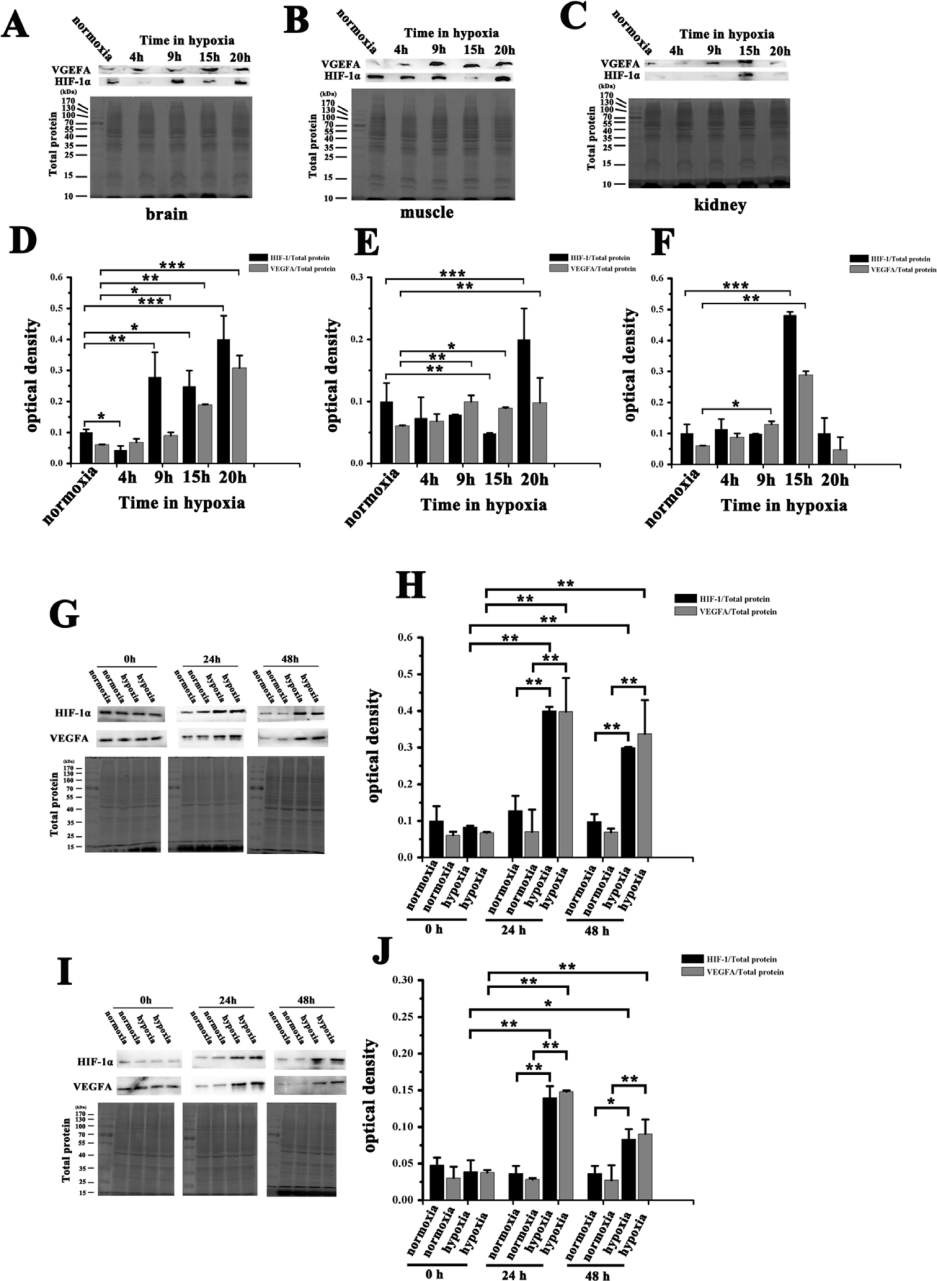
Expression of HIF-1α and VEGFA in NMRs and HSCs from NMRs or mice before and after hypoxia Western blot detected HIF-1α and VEGFA protein expression level in NMRs (**A**) brain, (**B**) muscle and (**C**) kidney after hypoxic stress for 4, 9, 15 or 20 h. Band density analysis of HIF-1α and VEGFA protein expression and expression patterns in (**D**) brain, (**E**) muscle and (**F**) kidney after hypoxia. Western blot analysis of the expression of HIF-1α and VEGFA in HSCs from (**G**, **H**) NMRs or (**I**, **J**) mice before and after hypoxic exposure for 24 h or 48 h. Total protein stain gel image was used as a control. Data represented as mean ± SEM of triplicate measurements obtained from three different animals. ^*^p < 0.05, ^**^p < 0.01, ^***^p < 0.001.

We determined the expression of HIF-1α and VEGFA protein in brain, muscle and kidney of NMRs exposed to acute hypoxia (5% O_2_) for 4, 9, 15 or 20 h. HIF-1α protein showed a rising trend after an significant decline in brain (at 4 h) and muscle (at 15 h) tissue, whereas VEGFA protein level showed a slow rise and reached a significant level (*p* < 0.05) at 9 h (Figure 2A, 2B, 2D, 2E). HIF-1α and VEGFA in kidney tissue exhibited a similar pattern that rose to a maximum at 15 h and then decreased (Figure 2C, 2F).

### Hypoxia exacerbated the upregulation of HIF-1α in NMR HSCs compared to mouse HSCs

HIF-1α and VEGFA protein levels were assessed by Western blot in HSCs from NMRs or mice before and after hypoxic exposure for 24 or 48 h. Compared with the normoxic group, HIF-1α and VEGF protein levels in both hypoxic NMR and mouse HSCs rose significantly at 24 h (Figure 2G, 2I). With prolonged hypoxia, HIF-1α protein declined following its maximum at 24 h in NMRs (HIF-1α /total protein = 0.082 at 0 h, 0.399 at 24 h, and 0.299 at 48 h) and mouse HSCs (HIF-1α /total protein = 0.038 at 0 h, 0.129 at 24 h, and 0.072 at 48 h). These results demonstrate that NMRs displayed a higher level and a stronger upregulation of HIF-1α protein following hypoxia than mice. Consistent with whole-tissue data, NMRs have a higher levels of endogenous HIF-1α expression (0.082) than mice (0.038) at the cellular level.

### Hypoxia increased apoptosis in mouse, but not NMR HSCs

**Figure 3:**
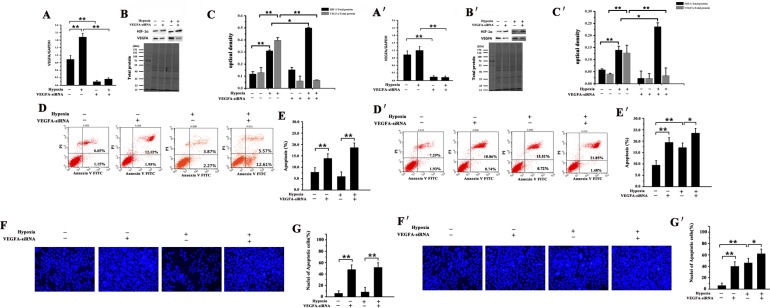
Effect of VEGFA-siRNA on HIF-1α and VEGFA expression and apoptotic level in NMR and mouse HSCs Real-time PCR analysis of VEGFA mRNA levels in HSCs from (**A**) NMRs and (**A′**) mice transfected with VEGFA-siRNA for 24 h before and after hypoxic treatment. GAPDH was used as an internal reference. Detection of VEGFA and HIF-1α protein in (**B**) NMR and (**B′**) mouse HSCs after transfection with VEGFA-siRNA for 48 h under normoxic or hypoxic conditions. Statistical analysis illustrates VEGFA and HIF-1α protein expression in (**C**) NMR and (**C′**) mouse HSCs after transfection with VEGFA-siRNA for 48 h. Both (**D**) NMR and (**D′**) mouse HSCs were transfected with VEGFA-siRNA or NC-siRNA for 48 h, and apoptosis was measured by flow cytometry. Statistical analysis illustrates apoptosis of (**E**) NMR and (**E′**) mouse HSCs after transfection with VEGFA-siRNA for 48 h. Fluorescence microscopy was used to observe the cell morphology following DAPI staining after transfection with VEGFA-siRNA for 48 h in (**F**) NMR and (**F′**) mouse HSCs (all panels, ×200). Quantification of nuclei of apoptotic cells in (**G**) NMR and (**G′**) mouse HSCs following DAPI staining. ^*^p < 0.05, ^**^p < 0.01.

**Figure 4:**
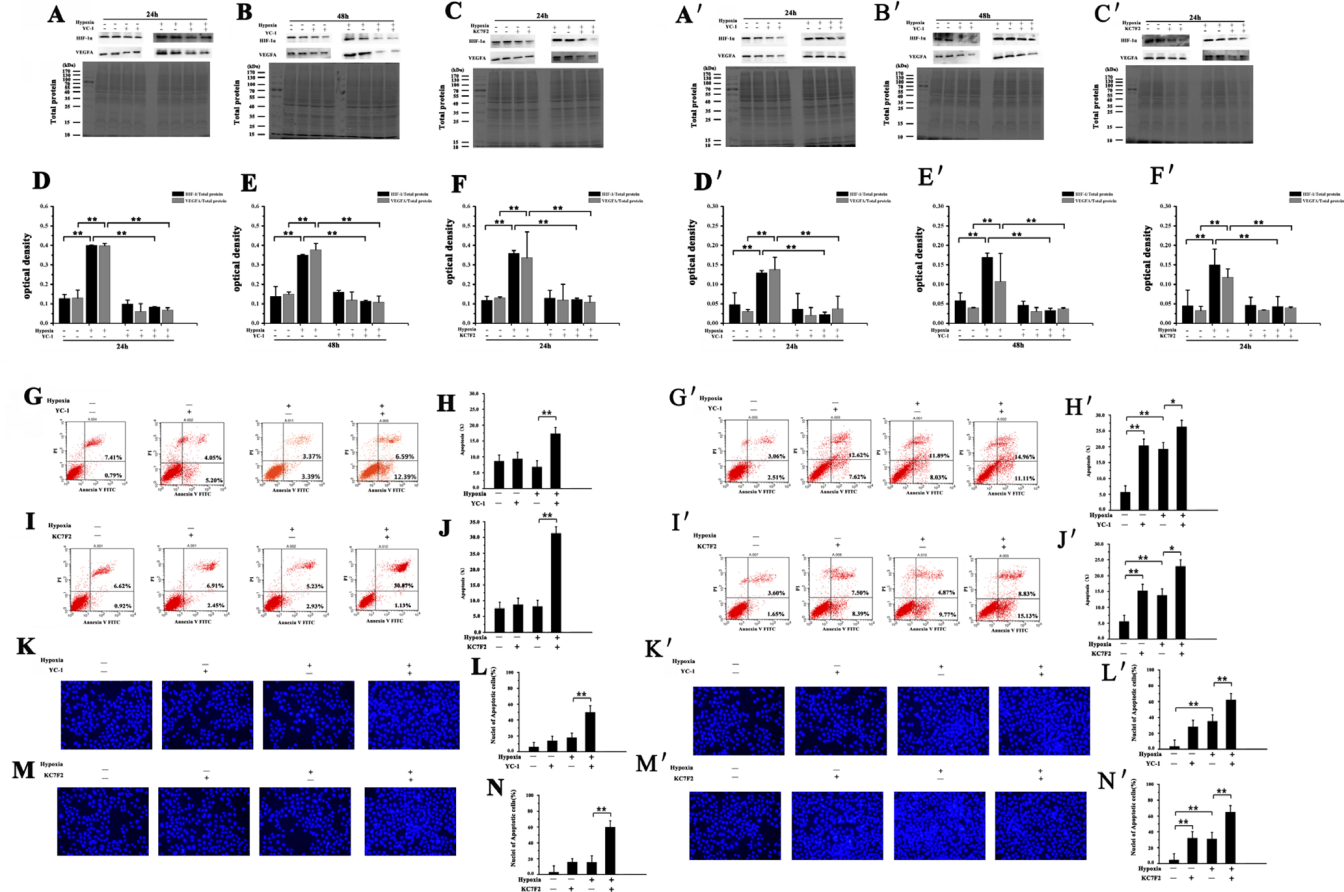
Effect of YC-1 or KC7F2 on HIF-1α and VEGFA expression and apoptosis in NMR or mouse HSCs HSCs were pretreated with 50 μM YC-1 or 50 μM KC7F2 with or without hypoxic exposure for 24 h or 48 h. (**A**, **B**, **C**) NMR and (**A′**, **B′**, **C′**) mouse cell lysates were subjected to Western blot analysis using VEGFA and HIF-1α specific antibodies. The intensity of the protein bands of three typical experiments of (**D**, **E**, **F**) NMR or (**D′**, **E′**, **F′**) mouse lysates was quantified using Quantity software. Total protein stain gel image was used as a control. Apoptosis of (**G**, **I**) NMR, (**G′**, **I′**) mouse cells was analyzed with annexin V and PI staining, and measured by flow cytometry. Statistical analysis of apoptosis of (**H**, **J**) NMR or (**H′**, **J′**) mouse cells following treatment with 50 μM YC-1 or 50 μM KC7F2 for 48 h. Cell morphology was observed using fluorescence microscopy following DAPI staining after treatment with 50 μM YC-1 or 50 μM KC7F2 for 48 h in (**K**, **M**) NMR or (**K′**, **M′**) mouse HSCs (all panels, ×200). Quantification analysis apoptotic cells in (**L**, **N**) NMR and (**L′**, **N′**) mouse HSCs following DAPI staining. Data are shown as the mean ± SEM of five independent experiments. ^*^p < 0.05, ^**^p < 0.01.

As shown in Figure 3 and 4, hypoxia induced a significant increase in apoptosis in mouse HSCs (9.2% to 16.23%), but not in NMR HSCs (the normoxic and hypoxic apoptosis is 7.8% and 6.14%, respectively). This suggests that NMRs cells are better adapted to hypoxic conditions than mouse cells.

### Inhibition of VEGFA increased HIF-1α and induced apoptosis in both NMR and mouse HSCs before and after hypoxia

We used VEGFA-siRNA to reduce VEGFA expression before and after hypoxic exposure in NMR and mouse cells. Compared with the control (NC-siRNA), VEGFA mRNA levels in the VEGFA-siRNA group decreased significantly at 24 h post-transfection both in NMR and mouse cells (*p* < 0.05, Figure 3A, 3A′) under normoxia or hypoxia. VEGFA protein in the VEGFA-siRNA group was significantly lower 48 h after transfection (Figure 3B, 3B′) in both species. Levels of apoptosis in the VEGF-siRNA group cells were increased significantly compared with the NC-siRNA group in both hypoxia and normoxia (Figure 3D, 3D′) consistent with DAPI staining results (Figure 3F, 3F′) in both species. However, apoptosis in the VEGF- siRNA + hypoxia group was not significantly higher than that of the siRNA inhibition group alone.

HIF-1α protein levels in the VEGFA-siRNA group were significantly higher than in the NC-siRNA group 48 h after transfection (Figure 3B, 3B′), indicating that inhibiting expression of VEGFA promoted accumulation of HIF-1α.

### Inhibition of HIF-1α downregulated VEGFA and induced marked apoptosis in NMR HSCs following hypoxia

We examined the protein levels of HIF-1α and VEGFA in NMR and mouse HSCs treated with 50 μM YC-1 or KC7F2 before and after hypoxic exposure for 24 h or 48 h. HIF-1α and VEGFA protein levels decreased significantly in the YC-1 or KC7F2 treated groups compared with untreated controls in both NMR and mouse cells (Figure 4A–4CA, 4A′–4C′), indicating that inhibition of HIF-1α lead to downregulation of VEGFA in both cell types.

We measured apoptosis in HSCs after treating with 50 μM YC-1 or KC7F2 before and after hypoxic exposure for 48 h. As shown in Figure 4G–4JG, 4G′–4J′, the ratio of apoptotic cells was significantly increased in both NMR and mouse HSCs following treatment with YC-1 or KC7F2 for 48 h (*p* < 0.05). However, compared with mouse HSCs (14.44% to 23.96%), NMR HSCs displayed marked increase in levels of apoptosis (8.16% to 32.0%) following inhibition of HIF-1α after hypoxia. HSCs treated with YC-1 or KC7F2 for 48 h displayed significant nuclear punctate, indicating the DNA fragmentation and confirming the increase of apoptosis (Figure 4K–4NK, 4K′–4N′). However, our results revealed no significant effect on apoptosis of NMR HSCs following inhibition of HIF-1α under normoxia.

### Inhibition of HIF-1α markedly decreased autophagy levels in NMR cells

**Figure 5:**
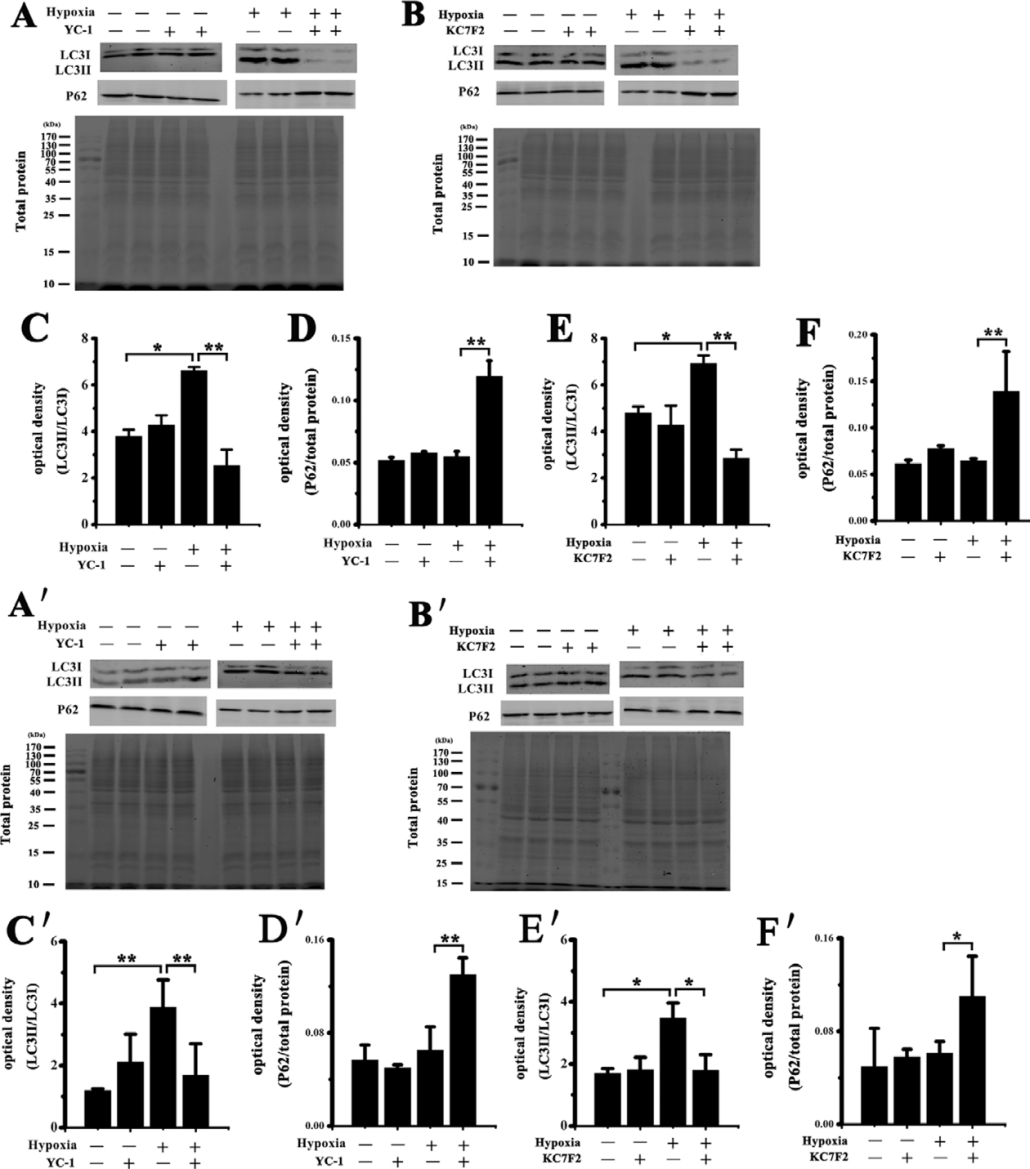
Western blot analysis of autophagy markers in NMR or mouse HSCs Western blot detected LC3 and p62 in (**A**, **B**) NMR and (**A′**, **B′**) mouse HSCs following treatment with 50 μM YC-1 or 50 μM KC7F2 for 24 h before and after hypoxic exposure. Total protein stain gel image was used as a control. Quantitative analysis of LC3 and p62 expression in (**C–F**) NMR and (**C′–F′**) mouse cells. Data are shown as the mean ± SEM. ^*^p < 0.05, ^**^p < 0.01.

The expression of autophagy markers (LC3, p62) was detected by Western blot following inhibition of HIF-1α using YC-1 or KC7F2 before and after hypoxic exposure in NMR and mouse HSCs. An increase in autophagy was observed in both NMR and mouse cells after hypoxic exposure. However, NMR HSCs displayed a higher level of autophagy (ratio LC3ΙΙ/LC3Ι = 9.6) than mouse HSCs (ratio LC3ΙΙ/LC3Ι = 4.9) following hypoxia. We further found that inhibition of HIF-1α lead to a more significant decrease of autophagy in NMR cells compared to mouse cells (Figure 5).

## DISCUSSION

More than 20% of the oxygen consumed by humans is utilized by the brain for the generation of ATP to facilitate the membrane potentials required for synaptic activity, energy utilization and supply [10, 11]. Metabolic deficits, including disruption in oxygen supply, along with the resulting energy deficit, is now thought to cause neuronal death through hypoxic neuronal injury. Several lines of evidence indicate that the identification of a potent target and its translation into an applicable therapy has been difficult to achieve from animal models. Some evidence supports the premise that hypoxic tolerant brains in suitable animal models can offer important strategies in developing new therapies. Subterranean burrow-dwelling mammals, such as the naked mole-rat (Heterocephalus glaber), successfully maintain normal activity and body composition for at least 80% of their lives. They display no obvious hypoxia-related increases in morbidity or mortality and survive in dark, damp environments with low oxygen levels and poor quality nutrition [12]. The hepatic stellate cells comprise approximately one-third of the nonparenchymal cell population and 15% of the total number of resident cells in a normal liver. This cell type is a remarkably versatile mesenchymal cell, vital to hepatocellular function and the liver's response to injury [13]. The naked mole rats are not a closed colony, but only a single female breeds in a colony. ICR mice are internationally outbred mice, and are sensitive to hypoxic conditions. Therefore, the outbred ICR mouse strain was used in this comparative study.

Since endotherms regulate their body temperature within a narrow range during tolerance to hypoxia, Drew *et al*. suggested that chronic hypoxia of fossorial life could contribute to adaptations in a given species and would play significant roles in their adaptive physiology [14]. Resistance to tissue hypoxia in the naked mole rat is most likely an adaptation at the genetic level given their ancestral exposure to chronic hypoxia. Indeed, hypoxia tolerance is also exhibited by adult naked mole rats that have never been exposed to hypoxia during development. Such adaptations included metabolic suppression, which constitutes a fundamental survival response to maintain tissue viability in during acute or chronic hypoxia. An early study by Buffenstein and Yahav revealed a physiological adaptation which acts as the molecular oxygen sensor and can be directly linked to genes regulation during tissue hypoxia in the naked mole-rat [15]. In a follow-up study, Shams *et al*. [16] found that the transcription of multiple genes, such as those coding for the vascular endothelial growth factor (VEGF), erythropoietin (EPO), hypoxia-inducible factor 1 (HIF-1), and many glycolytic enzymes participated in the adaptive strategy of another mole rat species (Spalax) in withstanding tissue hypoxia.

HIF-1α is a major activator of downstream genes, such as vascular endothelial growth factor (VEGF) during hypoxia. The present study assessed the role of HIF-1α and VEGFA in response to hypoxia in the naked mole rat. As the amount of VEGF correlates with the activity of neovascularization, immunoblot analysis of VEGF is a reliable method to predict angiogenesis in mammalian cells. In this study, we demonstrated that VEGFA protein was high in brains of NMRs and mice, with no significant difference between the two species. There are no significant differences in HIF-1α and VEGFA protein expression in the lungs of NMRs and mice. However, HIF-1α and VEGFA protein in the liver, muscle, and kidney from hypoxia-tolerant NMRs was significantly higher than in hypoxia-sensitive mice. In the heart tissue, there was a significant difference in VEGFA protein but not HIF-1α. Quantitative analysis of HIF-1α and VEGFA mRNA expression was conducted with RT–PCR. As observed in Western blot analysis, the expression of HIF-1α and VEGFA trended higher in the naked mole rat tissues compared to mouse tissues, including the brain, lung, heart, liver, kidney, and muscle (Figure 1D, 1E). These results demonstrated that tissues from NMRs expressed significantly more HIF-1α and VEGFA than mice. Furthermore, we also demonstrated that the expression of HIF-1α and VEGFA in the brain, muscle, and kidney of NMRs was altered following acute hypoxic stimulation. In addition, we measured HIF-1α and VEGFA protein levels in NMR or mouse HSCs before and after hypoxic exposure. Our data showed that there is an induction of HIF-1α or VEGFA in both NMR and mouse HSCs after hypoxia, but NMR HSCs displayed a higher level and a stronger upregulation of HIF-1α protein (HIF-1α /total protein = 0.082 at 0 h, 0.399 at 24 h, and 0.299 at 48 h) than did mouse HSCs (HIF-1α /total protein = 0.038 at 0 h, 0.129 at 24 h, and 0.072 at 48 h). These results suggest that NMRs have a stronger ability to actively enhance the HIF-1α - mediated response and survive severe hypoxia compared to mice.

The expression patterns of HIF-1α and VEGFA varied in tissues examined in this study. HIF-1α expression in brain and muscle tissue from NMRs was reduced after hypoxia, but VEGFA expression slowly increased over the time course of the experiment. This suggests that HIF-1 was not necessary for maintaining activation of VEGFA under hypoxic conditions. However, expression of HIF-1α and VEGFA in the kidney exhibited similar patterns (Figure 2). This suggests that HIF-1α and VEGFA have different expression patterns in different tissues, indicating that NMRs may have a unique regulatory mechanism after acute hypoxia. Previous studies found that inhibiting the metabolic rate of the brain to reduce oxygen consumption is a neuroprotective mechanism in perinatal stages and ischemic hypoxia [17]. The mRNA expression of more than 95% of genes is downregulated after hypoxic stimulus [18]. In this study, interesting differences in hypoxia responses were found by comparing different tissues, with the brain exhibiting the most hypoxic markers. HIF-1α protein levels in brain rose more quickly than in muscle or kidney in NMRs, suggesting that HIF-1-mediated responses to hypoxia in brain were faster. This finding demonstrates that HIF-1 may be partly responsible for maintaining normal neuronal function under hypoxic conditions [19].

Many conditions that impose stress on cells, including hypoxia, starvation, infections, and changes in secretory needs, challenge the folding capacity of the cell and promote endoplasmic reticulum stress. The accumulation of misfolded or unfolded proteins indicates problems in cellular homeostasis that frequently end in cell death [20]. Hypoxia inducible factor-1α (HIF-1α) is a critical transcription factor activated under this adverse condition, and can activate the transcription of genes controlling metabolism, cell survival, death, and angiogenesis. Considering the high levels of endogenous HIF-1α and its activation after hypoxia, we further explored the role of HIF-1α and VEGFA on the cell survival in the naked mole rat. We found that hypoxia induced a significant increase in apoptosis of mouse HSCs but not NMR HSCs (Figures 3 and 4). This suggests that NMR cells are better adapted to hypoxic conditions compared to mouse cells. Then, we further detected apoptosis following inhibition of HIF-1α in NMR and mouse HSCs treated with a HIF-1α inhibitor (YC-1 or KC7F2) under the normoxic or hypoxic condition. Our results showed a significantly increased level in apoptosis both in NMR and mouse cells following inhibition of HIF-1α under hypoxic conditions. However, compared with mouse HSCs (14.44% to 23.96%), NMR HSCs displayed a far greater increase in apoptosis (8.16% to 32.0%) following inhibition of HIF-1α under the hypoxic environment (Figure 4). We found that knockdown of VEGFA resulted in an increase in apoptosis in both NMR and mouse HSCs before (NMR: 7.8% to 14.07%; mouse: 9.22% to 19.6%) and after (NMR: 6.14% to 18.18%; mouse: 16.23% to 23.33%) hypoxia, indicating that VEGFA transcription is important for cell survival under normoxic or hypoxic conditions. We assumed that VEGFA was not necessary to the naked mole rat tolerance to hypoxia and its high expression was a result of upregulation following HIF-1α activation. All of the above results revealed that NMRs possess powerful resistance to hypoxic conditions through control of apoptosis and is likely regulated by HIF-1α activation.

Hypoxia-induced autophagy can serve as a mechanism to turn over nutrients, to mitigate the adverse condition and then potentially avoid cell death [21]. Our previous study reported a marked increase in autophagic activity and a slight increase in apoptosis in NMR HSCs following H_2_O_2_ treatment, suggesting NMRs utilize autophagy to prevent excessive apoptosis and maintain their functionality [22]. HIF-1α activation has been linked directly to autophagy and the clearing of necrotic cells. Based on the HIF-1α activation we discovered, we further measured the levels of autophagy in NMR or mouse HSCs before and after hypoxic treatment. Recently, an increase in autophagy was observed in both NMR and mouse cells after hypoxic exposure. However, NMR HSCs displayed higher levels of autophagy (LC3ΙΙ/LC3Ι = 9.6) than mouse HSCs (LC3ΙΙ/ LC3Ι = 4.9) under hypoxic conditions (Figure 5). We further demonstrated that inhibition of HIF-1α lead to a more significant decrease of autophagy in NMR cells compared to mice. Autophagy is the cellular process that mediates the lysosomal degradation of long-lived cytoplasmic proteins; the amino acids produced during autophagy can be used to synthesize proteins that are important for the organism's adaptation to nutrient-deficient environments. Taken together, we suggest that NMR cells have a greater capacity in utilizing autophagy to recycle cellular materials during hypoxia. In addition, autophagy can also be indicative of cell death, so there was another possibility that high autophagic levels could also resulted in cell death when they had suffered excessive damage during hypoxia.

In conclusion, our results suggest that accumulation of HIF-1α protein in tissues of hypoxia-tolerant NMRs was higher than in hypoxia-sensitive mice. Furthermore, hypoxia-induced HIF-1α expression patterns were different in different tissues. We also report that inhibition of HIF-1α induced a greater increase in apoptosis in NMR HSCs compared to mouse HSCs under hypoxic conditions. However, blocking VEGFA transcription induced apoptosis before and after hypoxia in both naked mole rat and mouse cells, suggesting that high expression of VEGFA may be a result of upregulation following HIF-1α activation. Furthermore, NMR cells had a greater capability in utilizing autophagy to degrade damaged organelles during hypoxia compared to mouse cells. Future studies will explore the mechanism of HIF-1 facilitating the survival of NMRs HSCs under hypoxic conditions.

## MATERIALS AND METHODS

### Animals

Adult ICR mice (eight weeks old) were purchased from SLAC Laboratory Animal Co., Ltd. (Shanghai, China). NMRs were purchased from the Department of Zoology at the University of Cape Town and raised at the Laboratory Animal Center of the Second Military Medical University. All animals were housed at their optimum temperature (mice at 23°C, NMRs at 28°C). To test for acute hypoxic stress (5% O_2_), adult NMRs of a similar weight (51.61 ± 7.9 g) were placed in an 81 × 58 × 121 cm^3^ chamber divided into three compartments and a gas mixture was delivered at 3.5 L/min. A total of five mice and five NMRs were used for basal measurements, and fifteen NMRs were used for experimental interventions. Treatment of animals at all times was consistent with current regulation GB14925-2001: Laboratory animal requirements of environment and housing facilities (Chinese version).

### Tissue preparation

Animals were euthanized by pentobarbital overdose. Brain, lung, heart, liver, muscle and kidney tissues were quickly excised and put into liquid nitrogen. The tissue samples were cut into small pieces, and total protein was extracted using protein extraction reagent (Wuhan Boster Biotech, Wuhan, China) and a tissuelyser (Shanghai Jingxin Industrial Development Co., Ltd). Samples were used in the subsequent polyacrylamide gel electrophoresis.

### RNA extraction and real-time PCR to detect gene expression

Real-time PCR was performed to detect HIF-1α and VEGFA mRNA expression in NMR and mouse brain, lung, heart, liver, kidney and muscle. RNA was extracted with Trizol reagent according to the manufacturer's instructions. Total RNA (500 ng) was transcribed using the TIANScript cDNA First-Strand Kit (Tiangen Biochemical Technology Co., Ltd., Beijing, China). mRNA levels were measured by the StepOne Plus Real-time PCR Detection System (Applied Biosystems, Warrington, UK) and SYBR^®^ Green Master Mix (Applied Biosystems). Glyceraldehyde-3-phosphate dehydrogenase (GAPDH) was used as a loading control. Primers for detection of NMRs or mice HIF-1α and VEGFA mRNA is shown in Table 1. mRNA expression was calculated using the ΔΔCt method. Primers were directed against conserved regions of the genes to allow for comparison of expression between NMRs and mice.

**Table 1:** Primer Sequences used for qRT-PCR

Genes	Primer	Sequence
NMR HIF-1α	Forward	5′-GAGGTGGATATGTCTGGGTTG -3′
	Reverse	5′- AGGGAGAAAATCAAGTCGTGC -3′
NMR VEGFA	Forward	5′-TGCCCTTGGTGGGGTTTG-3′
	Reverse	5′-CGAGACGCTGGTGGACATC-3′
NMR GAPDH	Forward	5′-TGGAGAAAGCGGCCAAATAC-3′
	Reverse	5′-AAAGGTGGAAGAGTGGGTG-3′
Mouse HIF-1α	Forward	5′-GGGGAGGACGATGAACATCAA-3′
	Reverse	5′-GGGTGGTTTCTTGTACCCACA-3′
Mouse VEGFA	Forward	5′-AAGCAGATGGTCAAATCG-3′
	Reverse	5′-GGGGCATTAGAAGGTTGT-3′
Mouse GAPDH	Forward	5′-CATGGCCTTCCGTGTTCCTA-3′
	Reverse	5′- GCGGCACGTCAGATCCA-3′

### Cell culture and hypoxic stress

Primary hepatic stellate cells (HSCs) were isolated from NMRs or mice according to methods reported previously [23], and were cultured in DMEM medium containing 10% FBS, 100 units/ml penicillin and 100 mg/ml streptomycin at 5% CO_2_ and 20% O_2_ and 35°C as previously optimized. For hypoxic conditions, HSCs were maintained under 90% N_2_, 5% CO_2_, and 5% O_2_ at 35°C. All cells were used between passages 5–10.

### Small interfering RNA transfection

RNA interference (RNAi) is a tool utilizing double-stranded RNA induced sequence-specific gene silencing to rapidly block the expression of the genes. Small interfering RNA (siRNA) plays a vital role in RNAi [24]. siRNA was synthesized by Biomics Biotechnology Co., Ltd. (Nantong, China). HSCs were seeded in six well plates with complete medium under normoxic or hypoxic conditions. HSCs were transfected with 50 nM siRNA for 6 h according to the manufacturer's instructions. The transfection mixture was removed, and HSCs were cultured with a complete medium for another 24 h or 48 h. VEGFA mRNA expression was detected by real-time PCR after transfecting for 24 h. VEGFA or HIF-1α protein expression levels were measured by western blot after 48 h. The VEGF-siRNA sequences are shown in Table 2.

**Table 2:** VEGF-siRNA sequence used for RNA interference

Genes	Primer	Sequence
NMR VEGFA-siRNA	Forward	5′-CCACUGAGGAGUUCAACAUdTdT-3′
	Reverse	5′-AUGUUGAACUCCUCAGUGGdTdT-3′
Mouse VEGFA-siRNA	Forward	5′-GGAUGUCGUACCUGCUAUAdTdT-3′
	Reverse	5′-UAUAGCAGGUACGACAUCCdTdT-3′

### Apoptosis analysis

HSCs were seeded in six well plates and grown to 60–70% confluence under normoxic or hypoxic conditions. Cells were then treated with 50 μM YC-1, 50 μM KC7F2 or VEGFA-siRNA for 24 h or 48 h. Cells were trypsinized and washed twice with ice-cold PBS and incubated with annexin V-fluorescein isothiocyanate and propidium iodide (Nanjing KGI biotechnology, China) at 37°C in the dark for 30 min. Apoptosis was measured by flow cytometry (Becton Dickinson, USA). At the same time, apoptotic cells were also detected based on morphological changes with 4,6-amidino-2-phenylindole (DAPI) staining, and cells with shrunken or fractured nuclei were judged to be apoptotic.

### Western blot analysis

Adult NMRs were housed in 5% O_2_ for 4, 9, 15 or 20 h. HSCs isolated from NMRs or mice were seeded in six well plates and incubated at 5% O_2_, 5% CO_2_, 90% N_2_ at 35°C for 24 h or 48 h. After 24 h, total protein was extracted and protein was separated by polyacrylamide gel electrophoresis and transferred to PVDF membrane. Blots were blocked overnight at 4°C and incubated with primary antibody at 4°C for 2 h (HIF-1α, LC3: 1:1000, Abcam, Cambridge, UK; VEGFA, p62: 1:1000, Cell Signaling Technology, Shanghai, China). Blots were washed and incubated with the appropriate secondary antibody. Bands were visualized with the Kodak Gel Logic 4000 R Imaging System (Carestream, USA). We conducted comparative expression analyses of endogenous HIF-1α expression between species using a common loading control (β-actin). All quantitative Western blot experiments in hypoxia-treated species were normalized to total protein. Total protein analysis was performed by Coomassie blue staining as previous study reported [25].

### Statistical analysis

We used SPSS17.0 statistical software for statistical analysis of the data. All experimental data were derived from three independent experiments except where indicated otherwise. Statistical significance was assessed by Student's *t*-test. *P* < 0.05 was considered statistically significant.

## Abbreviations

NMR: naked mole rat
HIF-1: Hypoxia inducible factor-1
VEGFA: vascular endothelial growth factor A
EPO: erythropoietin
HSCs: hepatic stellate cells
ATP: adenosine triphosphate
GAPDH: Glyceraldehyde-3-phosphate dehydrogenase.

## References

[1] Lewis KN, Soifer I, Melamud E, Roy M, McIsaac RS, Hibbs M, Buffenstein R (2016). Unraveling the message: insights into comparative genomics of the naked mole-rat. Mamm Genome.

[2] Buffenstein R, Jarvis JU (2002). The naked mole rat--a new record for the oldest living rodent. Sci SAGE KE.

[3] Park TJ, Reznick J, Peterson BL, Blass G, Omerbasic D, Bennett NC, Kuich P, Zasada C, Browe BM, Hamann W, Applegate DT, Radke MH, Kosten T (2017). Fructose-driven glycolysis supports anoxia resistance in the naked mole-rat. Science.

[4] Gesser H, Johansen K, Maloiy GM (1977). Tissue metabolism and enzyme activities in the rodent Heterocephalus glaber, a poor temperature regulator. Comp Biochem Physiol B.

[5] Pamenter ME, Dzal YA, Milsom WK (2015). Adenosine receptors mediate the hypoxic ventilatory response but not the hypoxic metabolic response in the naked mole rat during acute hypoxia. Proc Biol Sci.

[6] Semenza GL (2017). A compendium of proteins that interact with HIF-1alpha. Exp Cell Res.

[7] Mazure NM, Chen EY, Laderoute KR, Giaccia AJ (1997). Induction of vascular endothelial growth factor by hypoxia is modulated by a phosphatidylinositol 3-kinase/Akt signaling pathway in Ha-ras-transformed cells through a hypoxia inducible factor-1 transcriptional element. Blood.

[8] Semenza GL, Jiang BH, Leung SW, Passantino R, Concordet JP, Maire P, Giallongo A (1996). Hypoxia response elements in the aldolase A, enolase 1, and lactate dehydrogenase A gene promoters contain essential binding sites for hypoxia-inducible factor 1. J Biol Chem.

[9] Kim EB, Fang X, Fushan AA, Huang Z, Lobanov AV, Han L, Marino SM, Sun X, Turanov AA, Yang P, Yim SH, Zhao X, Kasaikina MV (2011). Genome sequencing reveals insights into physiology and longevity of the naked mole rat. Nature.

[10] Herculano-Houzel S (2011). Scaling of brain metabolism with a fixed energy budget per neuron: implications for neuronal activity, plasticity and evolution. PLoS One.

[11] Nagase M, Takahashi Y, Watabe AM, Kubo Y, Kato F (2014). On-Site Energy Supply at Synapses through Monocarboxylate Transporters Maintains Excitatory Synaptic Transmission. J Neurosci.

[12] Johansen K, Lykkeboe G, Weber RE, Maloiy GM (1976). Blood respiratory properties in the naked mole rat Heterocephalus glaber, a mammal of low body temperature. Respir Physiol.

[13] Friedman SL (2008). Hepatic stellate cells: protean, multifunctional, and enigmatic cells of the liver. Physiol Rev.

[14] Drew KL, Buck CL, Barnes BM, Christian SL, Rasley BT, Harris MB (2007). Central nervous system regulation of mammalian hibernation: implications for metabolic suppression and ischemia tolerance. J Neurochem.

[15] Buffenstein R, Yahav S (1991). Is the naked mole-rat Hererocephalus glaber an endothermic yet poikilothermic mammal?. J Therm Biol.

[16] Shams I, Avivi A, Nevo E (2004). Hypoxic stress tolerance of the blind subterranean mole rat: expression of erythropoietin and hypoxia-inducible factor 1 alpha. Proc Natl Acad Sci U S A.

[17] Nathaniel TI (2008). Brain-regulated metabolic suppression during hibernation: a neuroprotective mechanism for perinatal hypoxia-ischemia. Int J Stroke.

[18] Hochachka PW, Buck LT, Doll CJ, Land SC (1996). Unifying theory of hypoxia tolerance: molecular/metabolic defense and rescue mechanisms for surviving oxygen lack. Proc Natl Acad Sci U S A.

[19] Kim SJ, Sung MS, Heo H, Lee JH, Park SW (2016). Mangiferin Protects Retinal Ganglion Cells in Ischemic Mouse Retina via SIRT1. Curr Eye Res.

[20] Iurlaro R, Munoz-Pinedo C (2016). Cell death induced by endoplasmic reticulum stress. FEBS J.

[21] Qiu Y, Li P, Ji C (2015). Cell Death Conversion under Hypoxic Condition in Tumor Development and Therapy. Int J Mol Sci.

[22] Zhao S, Luo H, Kan G, Zhao Y, Lin L, Tang Q, Yu C, Sun W, Cai L, Cui S (2014). The protective role of autophagy in Heterocephalus glaber hepatic stellate cells exposed to H2O2 or nutritional stress. Cell Physiol Biochem.

[23] Ramm GA (1998). Isolation and culture of rat hepatic stellate cells. J Gastroenterol Hepatol.

[24] Xiao X, Gang Y, Wang H, Wang J, Zhao L, Xu L, Liu Z (2015). Double-stranded RNA transcribed from vector-based oligodeoxynucleotide acts as transcription factor decoy. Biochem Biophys Res Commun.

[25] Eaton SL, Roche SL, Llavero Hurtado M, Oldknow KJ, Farquharson C, Gillingwater TH, Wishart TM (2013). Total protein analysis as a reliable loading control for quantitative fluorescent Western blotting. PLoS One.

